# Surreptitious Adversarial Examples through Functioning QR Code

**DOI:** 10.3390/jimaging8050122

**Published:** 2022-04-22

**Authors:** Aran Chindaudom, Prarinya Siritanawan, Karin Sumongkayothin, Kazunori Kotani

**Affiliations:** 1 Division of Transdisciplinary Science, Japan Advanced Institute of Science and Technology, Nomi 923-1292, Japan; ikko@jaist.ac.jp; 2 School of Information Science, Japan Advanced Institute of Science and Technology, Nomi 923-1292, Japan; 3 Department of Computer Engineering, Faculty of Engineering, Mahidol University, Nakhon Pathom 73170, Thailand

**Keywords:** adversarial QR, adversarial attack, deep learning, Convolutional Neural Networks

## Abstract

The continuous advances in the technology of Convolutional Neural Network (CNN) and Deep Learning have been applied to facilitate various tasks of human life. However, security risks of the users’ information and privacy have been increasing rapidly due to the models’ vulnerabilities. We have developed a novel method of adversarial attack that can conceal its intent from human intuition through the use of a modified QR code. The modified QR code can be consistently scanned with a reader while retaining adversarial efficacy against image classification models. The QR adversarial patch was created and embedded into an input image to generate adversarial examples, which were trained against CNN image classification models. Experiments were performed to investigate the trade-off in different patch shapes and find the patch’s optimal balance of scannability and adversarial efficacy. Furthermore, we have investigated whether particular classes of images are more resistant or vulnerable to the adversarial QR attack, and we also investigated the generality of the adversarial attack across different image classification models.

## 1. Introduction

Deep Learning (DL) is one of the most powerful technologies adopted by multiple commercial and scientific applications. It surpasses human performance in various tasks, including image recognition. However, as Deep Learning models develop in effectiveness and efficiency, the vulnerabilities of such models also become important to study and improve. Adversarial examples, a term to describe modified inputs that can manipulate the results of image classifier models [1,2], present a serious security challenge [3] for image classifier implementations, especially the models implemented for high-risk tasks such as facial recognition and autonomous driving systems. Various research studies have also proven that adversarial examples can be successfully used to attack models implemented in the physical world [4].

This research studies the efficacy and conspicuousness of adversarial examples against Convolutional Neural Networks (CNN)-based classification models by using an adversarial patch in the form of altered QR code images patched onto input images, creating adversarial examples that can deviate the weights of the machine learning model. On the one hand, there are existing research studies that displayed the adversarial examples’ effectiveness against object detection [5] and object classification [6] models. However, the adversarial patches in such research were obvious to human perception. This research will focus not only on improving the adversarial efficacy of the QR patches against various models but also on making the created QR patch scannable using a QR scanner to hide its true purpose as an adversarial example from human intuition. “Deep Learning” (DL) refers to a subset of machine learning techniques that make use of large volumes of data to create models that can identify useful patterns and their high-level semantics. Deep Learning, along with its subset Convolutional Neural Networks (CNNs), addresses the limitations of bottlenecks in computation [7] and high-dimensional input data processing [8] by creating a connection of multiple artificial neurons layers to describe the connection between multiple simple visual features, representing the whole object. Each neuron also maps high-dimensional inputs to output values using an activation function. A neural network can be generally described as follows:

(1) $$f\left(x\right)=f^{\left(k\right)}\left(f^{\left(2\right)}\left(f^{\left(1\right)}\left(x\right)\right)\right)$$

where *x* is an input image, and $f^{\left(i\right)}$ is a function of the *i*th network layer where *i* = 1, 2, …, *k.*

Research on adversarial examples usually simulate attacks against existing deep learning models such as Inception [9,10,11], ResNet [12], VGG [13], LeNet [14], and AlexNet [15]. Furthermore, such research make use of established computer vision datasets, including handwritten digits such as MNIST [16], image classification datasets such as CIFAR [17] and ImageNet [18]. The ImageNet dataset is also involved in the experiments of most adversarial approaches due to the enormous volume of the dataset, consisting of 14,196,122 images with 1000 classes.

The discussion of adversarial examples against machine learning models has been ongoing for over a decade, with previous targets often having handcrafted features such as intrusion detection and spam filters. Dalvi et al. [19] created a game simulating an adversary against a classifier in which both entities utilized cost-sensitive learning. Szegedy et al. [2] also proposed the L-BFGS method to adversarial examples using linear search, which was considered computationally expensive and impractical. Goodfellow et al. [1] then proposed the Fast Gradient Sign Method (FGSM) that adds a gradient sign to the cost function on each pixel, resulting in adversarial example training speed increases. The perturbations can be expressed as:

(2) $$\eta =\epsilon sign\left(\nabla_{x}J\left(\theta ,x,y\right)\right)$$

where η is the magnitude of the perturbation computed by using gradients from the back-propagation. Given an original image *x*, the generated adversarial example $x^{\prime }$ can be calculated by adding perturbation η on the original image $x^{\prime }=x+\eta$.

Sharif et al. [20] proposed printed adversarial eyeglasses as a method to attack facial recognition systems, where the attacker put on printed eyeglasses and presented their faces on a facial recognition system. However, the printed eyeglasses are considered inconspicuous to human intuition and can only be used against facial recognition scenarios where the testing environment had little variation in lighting scale and camera angles.

Eykholt et al. [5] also proposed perturbations to mimic graffiti on road signs with the focus on the conspicuousness of real-world adversarial attacks. The authors manually masked images of the target physical object in different angles, distances, and lighting conditions to increase the robustness of the adversarial example graffiti against varying distances and orientations. However, the applicability of this method in the physical world is limited, as the adversarial examples were generated with a series of individual graffiti pieces.

The concept of universal, real-world adversarial patch attacks against image classifiers was first introduced by Brown et al. [6], and it is based on the principle that image classifier models will output the image class based on the most salient patterns of the input. The adversarial patch was trained by applying the patch at a random position and scale to the input scene, which resulted in the input being detected as the trained patch’s target class. Xu et al. [21] also implemented the adversarial patch method by proposing a T-shirt pattern that will allow the person wearing it to evade a real-time person detector.

In this paper, we are interested in the conspicuousness to human intuition on the employment of the adversarial patches. We believe that making the adversarial patch readable by a QR scanner would alleviate the suspicions of its primary function as an adversarial tool.

There are studies on edge sensors with edge intelligence, which can scan images, conduct pre-processing such as edge detection, then use biological vision systems to reduce unnecessary areas of an image [22], and then apply complicated CNN on the sensors [23]. These edge sensors when used in conjunction with IR might be able to remove traditional adversarial patches from the scene. Due to the inherent nature of the QR code, removing the patch will also remove data contained in the QR code as well.

The integration of an adversarial example on a QR code can be viewed as an analogy to the watermarking concept, which is a very popular approach to hiding the information on the target signal. The method is very effective in inserting information while evading human perception. The concept of watermarking was applied with an adversarial attack by dispersing the adversarial pixels all over the target image, resulting in a successful attack with less noticeable patterns [24]. However, it is difficult to apply the method in practice due to the necessity of perturbing the pixels in specific locations all over the target image without noticeable changes. On the contrary, our proposed method can hide the attack underneath the QR code and free it from the location constraints.

The authors believe that an adversarial example’s intent to attack image classification models can be concealed by adding a scannable QR code to the patch, providing a stronger purpose for the patch for human acknowledgement. However, multiple factors must also be considered for this addition, such as the patch’s shape, the QR code’s ease of detection by the QR scanner, and the overall feasibility to employ this technique in the physical world, all of which are also investigated in this research.

The contributions of this work are as follows:

- Expand the adversarial patch concept by implementing the patch with a scan-ready QR code, in which this approach has yet to be explored to our knowledge.
- Utilize the QR code pattern to improve the feasibility and robustness of physical adversarial examples.
- Explore methods to allow adversarial examples to carry out additional information for usage in other applications, in addition to its primary purpose as an adversarial attack tool.
- Study possible differences between square-shaped and circular-shaped adversarial QR patches in terms of adversarial efficacy.
- Investigate the optimal brightness values for the adversarial QR patch’s dark parts in order to maximize the adversarial efficacy and the patch’s scannability toward a QR scanner.
- Investigate whether particular classes of images are more resistant or vulnerable to the QR patch attacks.
- Investigate the transferability of QR patch attacks on various deep learning models.

## 2. Materials and Methods

The general process to create the QR adversarial patches is based on [6], with adjustments made to accommodate the addition of the QR symbol into the adversarial patch.

### 2.1. Masked Patch Creation

**Figure 1:**
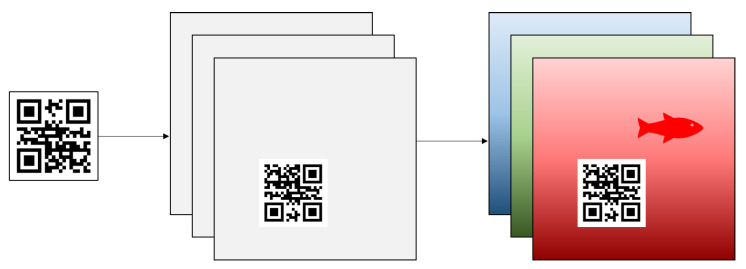
The QR patch masking with specified rotation and translation.

The adversarial patch application to an input image is represented by the operation A(p,q,x,r,l), where the patch application *A* is formed by translating patch *p* onto the input image *x* at location *l*, with *r* patch rotation. To begin the process of patch application, a matrix filled with only zeroes called a “mask image” will be created with the same size as the original input image *x*. A patch image *p* is then created as a QR symbol generated from a plain text or URL input string *q*, where the colors are represented with binary numbers where 0 represents black and 1 represents white colors. The generated patch is then rotated using randomized angle *r* between 0, 90, 180, or 270 degrees and then applied onto the mask image at random location *l*. Finally, the patch is applied onto the training images to create an adversarial example. Figure 1 displays the masked patch *p* creation process, where the black, white, and gray colors, respectively, represent zeros, random values and unassigned values.

### 2.2. Patch Training Process

Once the masked patch has been initialized, the next process is to apply the masked patch onto the training input image. For the initial patch weights, the black areas of the QR code symbol (i.e., the QR code’s contents) will be set to 0, since such an area would not be trained and must be kept. The adversarial QR patch $p^{\prime }$ is computed by maximizing the expectation of the [6]’s function as follows:

(3) $$p^{\prime }=\underset{p}{\mathrm{argmax}}E_{x∼X,r∼R,l∼L}\left[\log Pr\left(\hat{y}|A\left(p,q,x,r,l\right)\right)\right]$$

where $\hat{y}$ represents the target image classifier model’s confidence of the training image being the target class, where the patch operator *A* is applied over the distribution of *X* training set images. The patch location *l* and the randomized patch rotation *r* are also varied over the distribution of locations *L* and rotations *R* to improve the patch’s resiliency against varied placement locations and angles. Finally, *q* refers to the string used to generate the QR code symbol on the patch.

**Figure 2:**
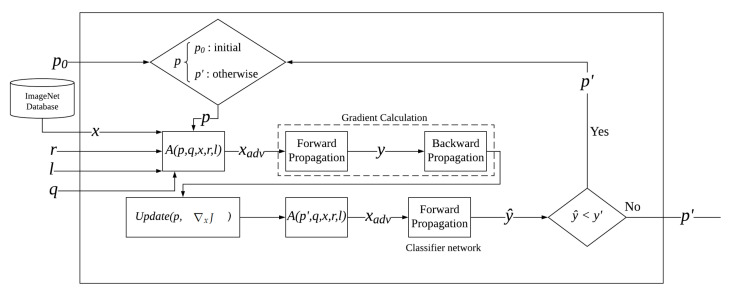
The architecture of the QR adversarial attack system for one training sample.

The QR adversarial example training process is displayed in Figure 2 where Equation (3) is applied during the process. The process starts with the forward and backward propagation on the adversarial example $x_{adv}$, where the gradient of the loss toward the target class obtained from backward propagation $\nabla_{x}J$ is used to update the patch in $Update(p,\nabla_{x}J)$. The update function can be expressed as:

(4) $$p_{new}^{\prime }=p^{\prime }-\nabla_{x}J$$

where $p^{\prime }$ is the current patch, and $p_{new}^{\prime }$ is the updated patch.

The updated patch $p_{new}^{\prime }$ is then reapplied onto the training image using the same operator A(p,q,x,r,l). The operator will recreate the adversarial example $x_{adv}$, which will be re-evaluated by the target image classifier model to obtain the training image’s confidence value of the target class $\hat{y}$. The training process will stop once the output prediction confidence value $\hat{y}$ exceeds the target confidence value $y^{\prime }$ or once the training process reaches the maximum amount of iterations specified from the parser before training. The final adversarial example $x_{adv}$ along with the adversarial QR patch $p^{\prime }$ is then returned from the process.

In addition, if the adversarial example $x_{adv}$ is successful in attacking the target model during the training process, it will be saved into the file system for further usage. Finally, as the objective of the training process is to alter the output prediction values from the target classifier network, the ground truth is not concerned with the backward propagation process of the target classifier network.

### 2.3. Circular-Shaped QR Adversarial Patch Implementation

It was observed in our empirical approach that the circular-shaped adversarial patches can retain their adversarial efficacy with each training iteration better than their square-shaped counterparts [25]. Therefore, an idea is implemented to apply the QR symbol onto a circular-shaped adversarial patch. The circular-shaped QR patch creation process is mostly homogeneous with the process to generate the square-shaped QR patches with several adjustments. Firstly, the QR code symbol is embedded at the size of 85% of the circular patch’s diameter instead of just using the generated QR image directly. This is to increase the area of adversarial perturbation while retaining the QR code symbol’s size compared to the square QR patches. Secondly, the size of the circular-shaped QR patch is slightly bigger compared to its squared counterpart (100 × 100 pixels for circular-shaped QR patch compared to 74 × 74 pixels for square QR patch) as the circular-shaped QR patch needs larger space to contain the same sized QR code as the square QR patch. Finally, the initial color of non-QR parts of the circular-shaped QR patch is randomized.

**Figure 3:**
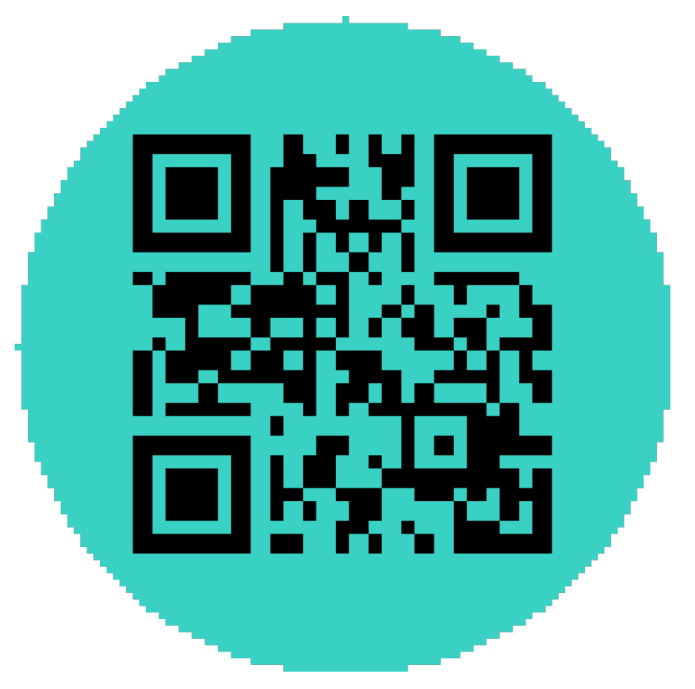
Initial circular QR patch.

**Figure 4:**
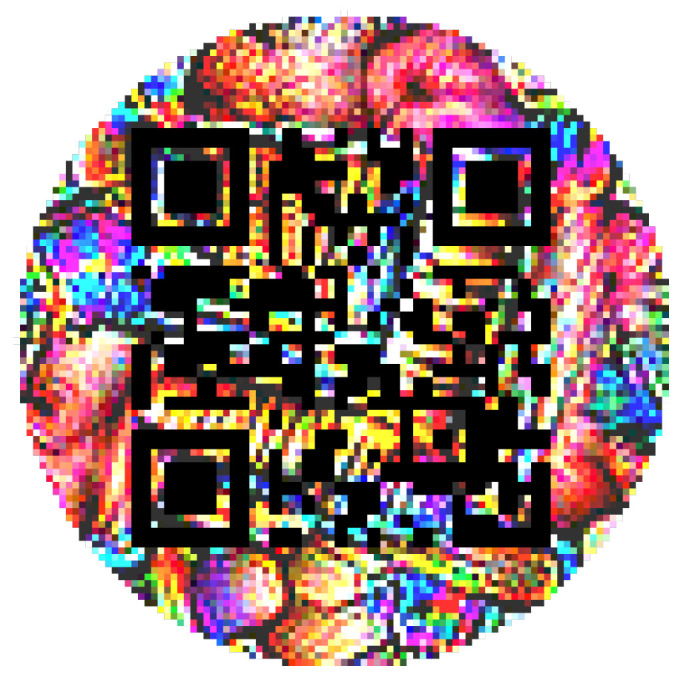
Trained circular QR patch.

**Figure 5:**
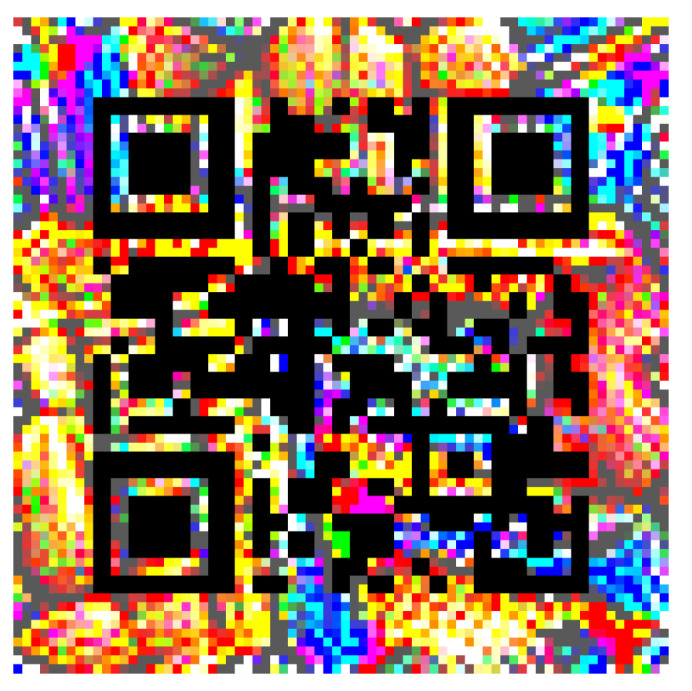
Trained square QR patch.

The initial circular-shaped QR patch is displayed in Figure 3, and the circular-shaped QR patch after the training process (τ = 50) is displayed in Figure 4. For comparison, Figure 5 displays the trained square-shaped QR patch from [26].

### 2.4. QR Code Visibility Improvement by Color Intensity Adjustment

The adversarial patch algorithm will sometimes produce black color patterns that reduce the visibility of the QR symbol on the patch. To address this issue, we adjust the patch’s black, non-QR-symbol areas to help distinguish between the QR symbol and the adversarial parts of the patch.

**Figure 6:**
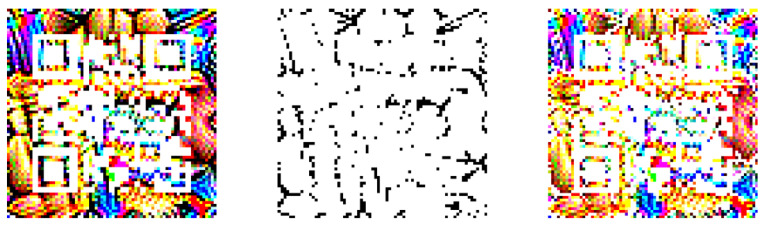
The process to make the trained QR patch visible to a QR scanner.

The process is done by creating a quiet zone surrounding the QR symbol by increasing the color intensity of the trained patch’s black (R:0, G:0, B:0) parts, making the patch more visible to a QR code scanner to read the patch’s QR contents. The τ addition function can be defined as:

(5) $$p_{out}^{\prime }\left(u,v\right)=\left\{\begin{array}{cc} p^{\prime }\left(u,v\right)+\tau , & \mathrm{if}\;p^{\prime }\left(u,v\right)=\left[0,0,0\right]\\ p^{\prime }\left(u,v\right), & \mathrm{otherwise} \end{array}\right.$$

where *u* and *v* represent the pixel’s coordinates on the patch $p^{\prime }$, and τ represents a small integer. Figure 6 displays the summary of processes to adjust the patch’s color intensity. The leftmost image displays the trained QR patch before the color intensity adjustment, the middle image displays only the black parts of the patch from the leftmost image, and the rightmost image displays the patch after the color intensity adjustments.

## 3. Results

### 3.1. Initialization and Parameter Settings

The patch training process begins with the initialization of the target image classification model, which will be instantiated from pre-trained image classification models. The default target image classification model is a convolutional neural network model known as InceptionV3 [10], which is also trained using the ImageNet ILSVRC2012 dataset [18] used to train the adversarial QR patch. In addition, the target image class and the threshold prediction value is also specified here to serve as a point to decide whether to move on to the next iteration or not. The default threshold value is set to 0.9, or 90% of the prediction confidence.

Next, the training image dataset is initialized by sampling 50,000 validation sets from the ImageNet ILSVRC2012 dataset, which is then split into training and testing sets. The images are then modified to optimize the training process by resizing them to 299 × 299 pixels (to prevent errors during the patch training and testing processes) and normalized using the dataset’s mean and standard deviation to improve time efficiency. Finally, the image is identified incorrectly by the pre-trained image classifier model by comparing the label of each image with the result from the pre-trained model.

### 3.2. Adversarial Efficacy of Advsersarial QR Patch

**Figure 7:**
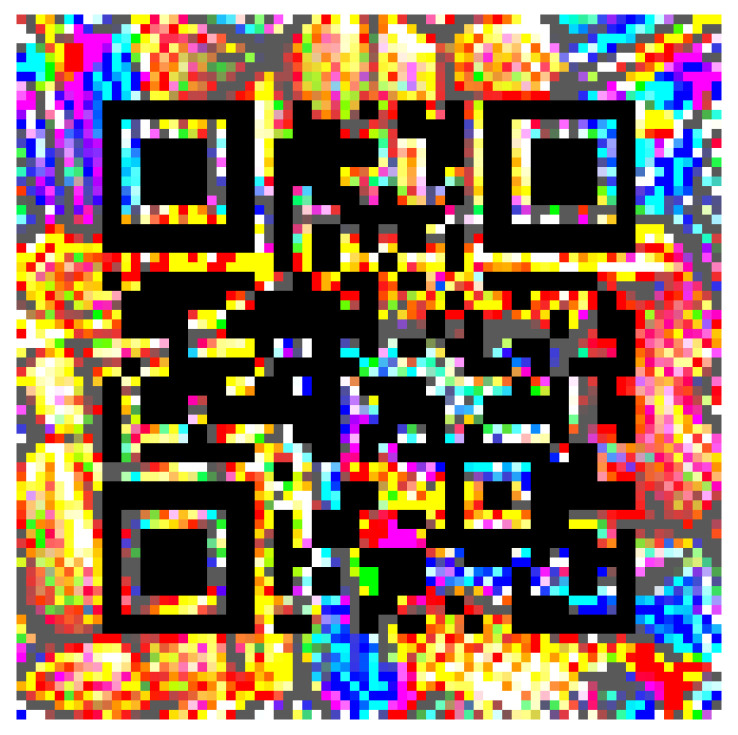
Square QR adversarial patch.

**Figure 8:**
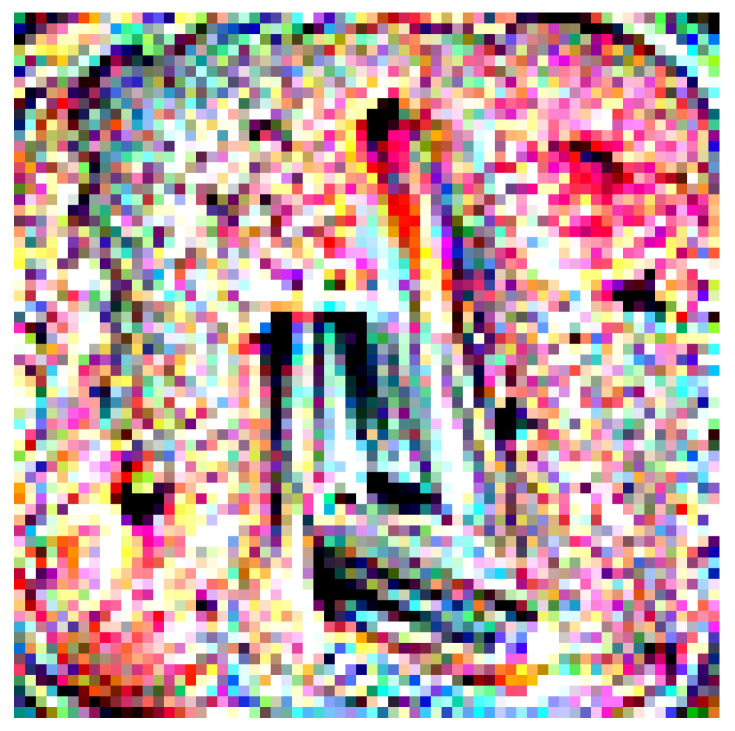
Square non-QR adversarial patch.

**Figure 9:**
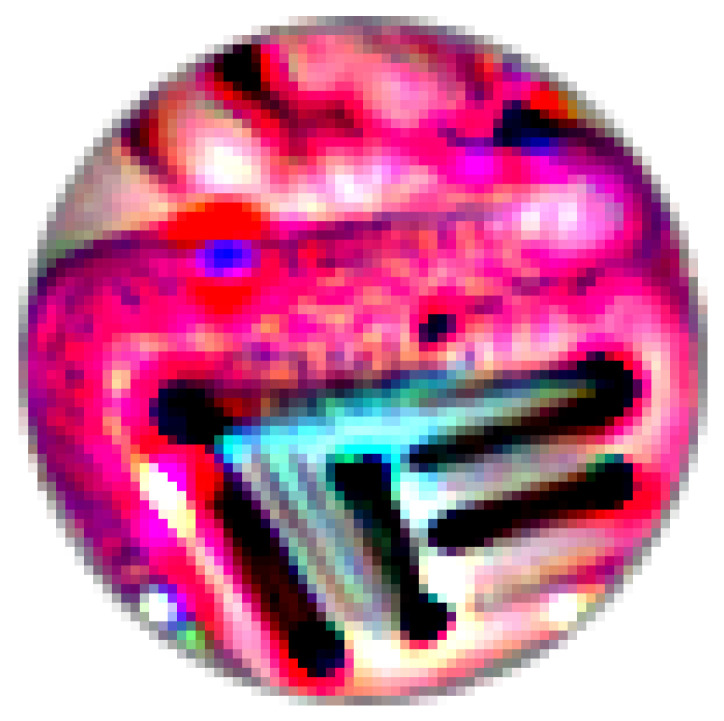
Circular non-QR adversarial patch.

**Figure 10:**
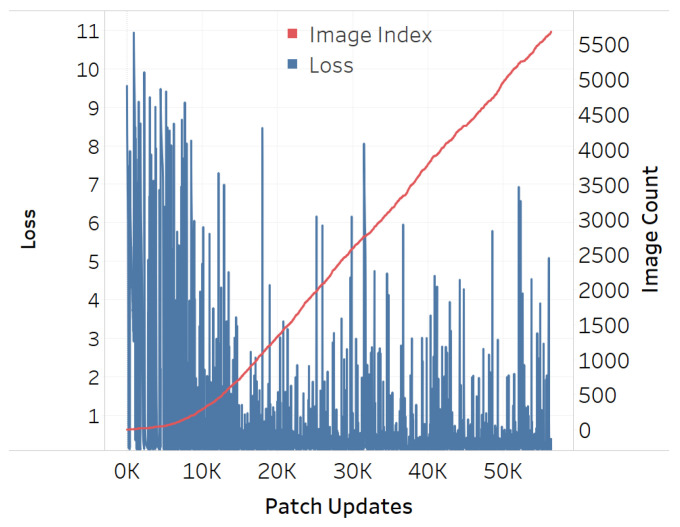
Training loss for QR-shaped patch.

**Figure 11:**
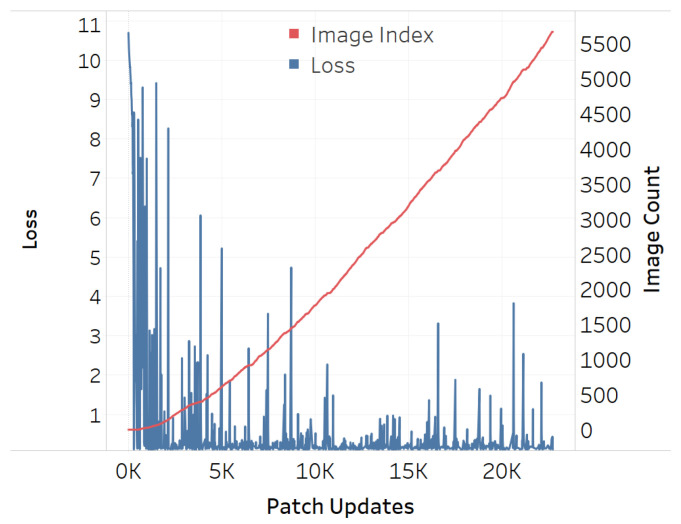
Training loss for square-shaped patch.

**Figure 12:**
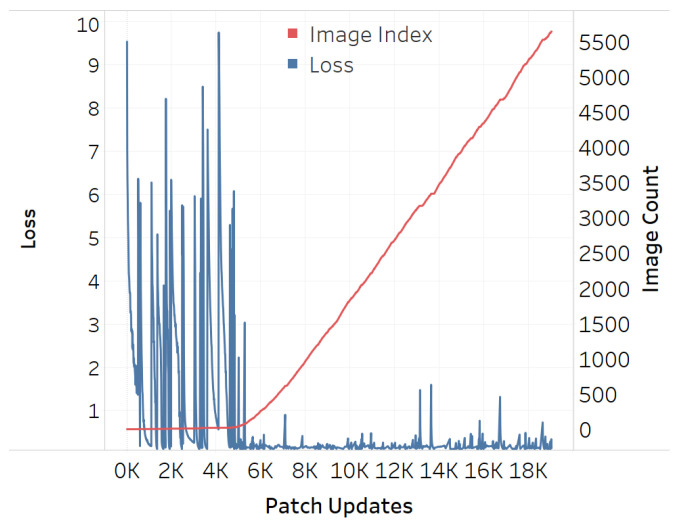
Training loss for circular-shaped patch.

The adversarial efficacy of the square-shaped adversarial QR patch shown in Figure 7 has been tested and compared against the non-QR square and circular-shaped shape patches shown in Figure 8 and Figure 9. The test was performed by recording loss values over patch updates calculated from randomly selected 6000 training images. Once the target class confidence is higher or equal to 0.9, or after 500 iterations have passed, the weights on the patch would then be carried over the next image to create the next attack iteration. The results displayed in Figure 10, Figure 11 and Figure 12 showed the loss values over patch updates, where the rightmost axis displays the index of the training image processed per each update iteration.

The results showed that all three types of patches show similar trends at an early stage. Later on, however, the circular patch displayed higher resistance to background scene changes as the loss values did not spike as much as its square counterparts, both QR and non-QR. A conjecture is made that the reason for the square shapes’ lower resistances is because the higher amount of edges and corners possess salient features hindering other features on the adversarial example. Every time a new input image is fed into the training system, such salient features would cause disruptions and lead to the spike of loss displayed in Figure 10, Figure 11 and Figure 12.

**Figure 13:**
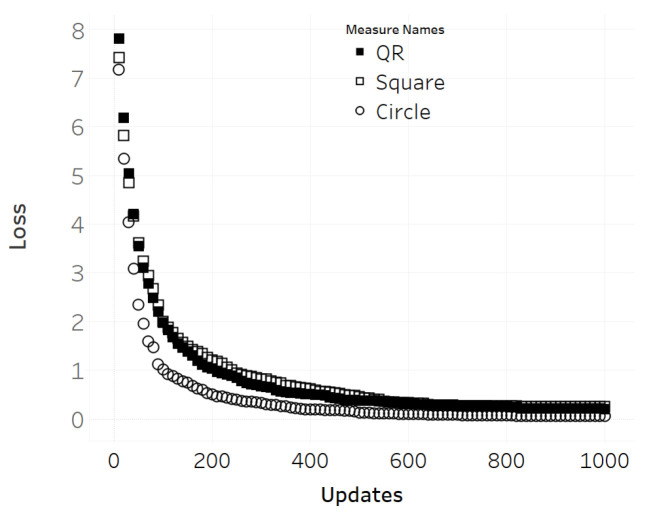
Loss per gradient 1000 update steps from initial state.

The next test performed is to evaluate the convergence speed of the patch update of each shape (Figure 13). This was done by measuring the average loss over 1000 update steps of the three different patch shapes (circle, non-QR square, QR square), in which the training images were sampled from the ImageNet dataset using the same seed for randomization to ensure that the same set of images was used for all three patch shapes during training. However, only 1000 iterations were performed for each scene due to computational power limitations. Figure 13 depicts the loss curve for each shape after the experiment, where the circular patch showed the fastest descent, followed by non-QR and QR square patches with negligible differences.

### 3.3. Variation of Adversarial QR Patch Conditions

**Figure 14:**
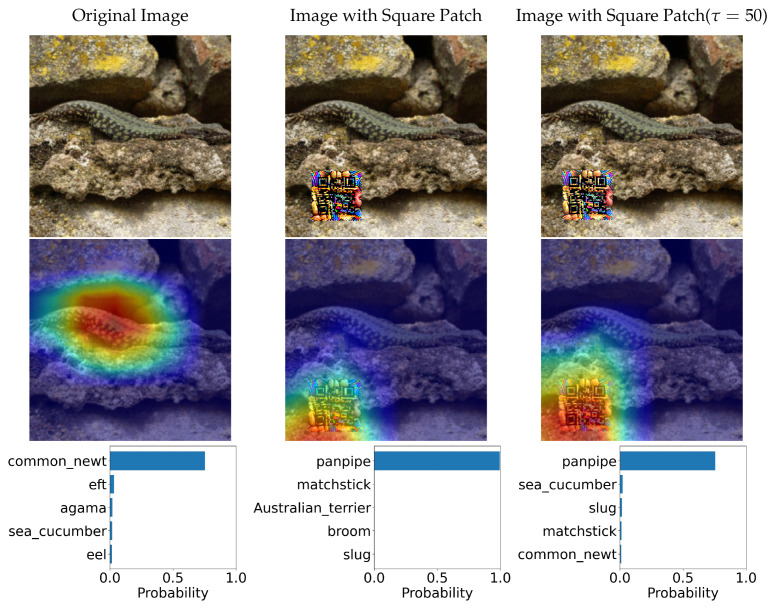
Classification predictions before and after application of the adversarial QR patch and the heatmap indicating saliency activation of the top predicted class (the color scheme is visualized from blue (**low**) to red (**high**)).

**Figure 15:**
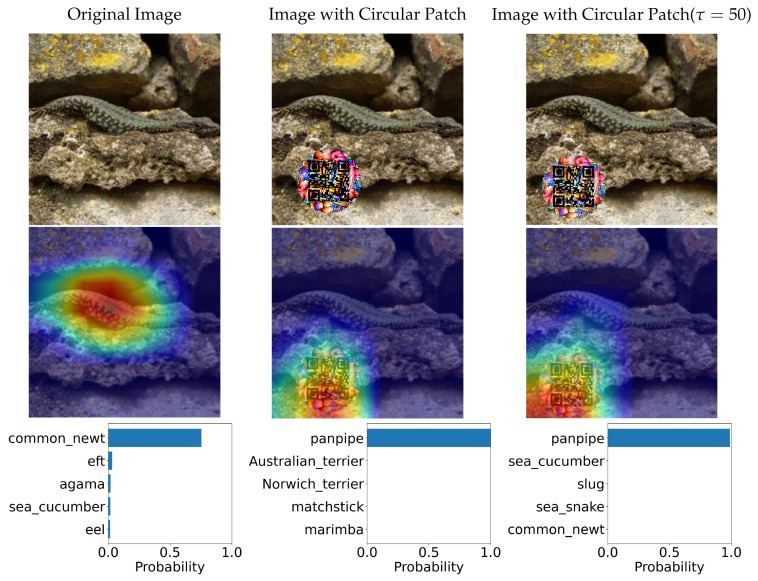
Classification predictions before and after application of the circular-shaped QR patch and the heatmap indicating saliency activation of the top predicted class (the color scheme is visualized from blue (**low**) to red (**high**)).

In this section, we tested two variations of Adversarial QR Patch conditions: *Shape of QR background* and *Color intensity* (τ). The examples of the results are shown in Figure 14 and Figure 15. Each figure shows the evaluation results of an image before and after applying the square-shaped adversarial QR patch and circular-shaped adversarial QR patch, including the prediction before and after applying color intensity adjustments to the patch. We also demonstrated the saliency activation of the top predicted class of each patch shape using GradCAM++ [27].

Furthermore, the circular-shaped QR patch’s adversarial efficacy after the color intensity adjustments with the τ value was then evaluated and compared to the square QR patch. The comparison was done by applying both patches onto 3492 ImageNet dataset images at the same location (u,v) = (80,160). Then, the average confidence value of the target class “Panpipe” was calculated with τ starting from 0 to 89. In addition, the circular-shaped QR patches were generated and trained using the same configurations to square patches to ensure accuracy of the comparison between both patch shapes.

**Figure 16:**
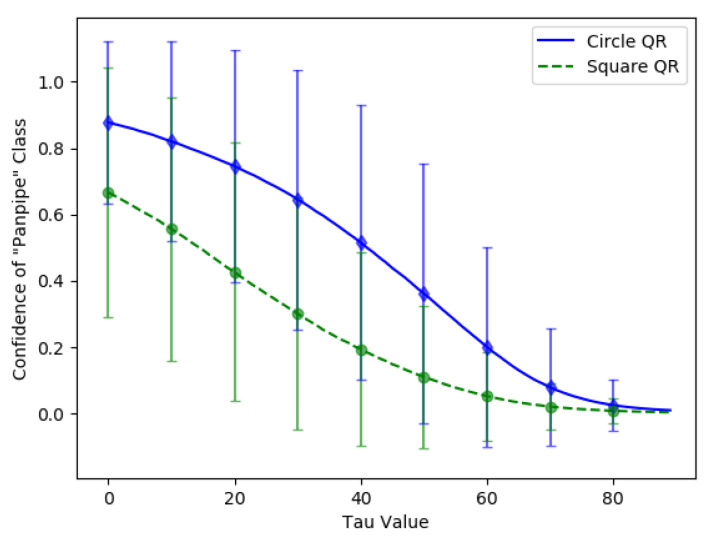
Average confidence values of the targeted “Panpipe” class for circular-shaped and square-shaped QR patches over the varying τ from 0 to 89.

The average confidence value of the “Panpipe” target class over τ updates from 0 to 89 can be found in Figure 16. The green line represents the average confidence value from the adversarial examples with the square QR patch, while the blue line represents the average score from the circular-shaped QR patch. The standard deviation of both patch shapes is also shown as the error bars of identical colors. The results showed that the average confidence value of the circular-shaped QR patch is higher than those of the square-shaped QR patch up to τ = 80 where the average confidence value became almost zero. We believe that the higher average adversarial efficacy and resistance to color intensity adjustments are due to the corners and edges of the square-shaped in a square QR patch which contain salient features disrupting other features in the image and reducing the square patch’s effectiveness. The effects are identical for both cases of QR and non-QR adversarial patches.

### 3.4. Trade-Off between Adversarial Efficacy and Scannability

Another experiment was performed to find the optimal τ value that can maximize the adversarial QR patch’s visibility to a QR scanner while maintaining the patch’s adversarial efficacy. The experiment was conducted by scanning the square and circular-shaped QR patches on a computer screen with a mobile phone camera (Samsung Galaxy S10) while also updating τ after every scanning attempt.

**Figure 17:**
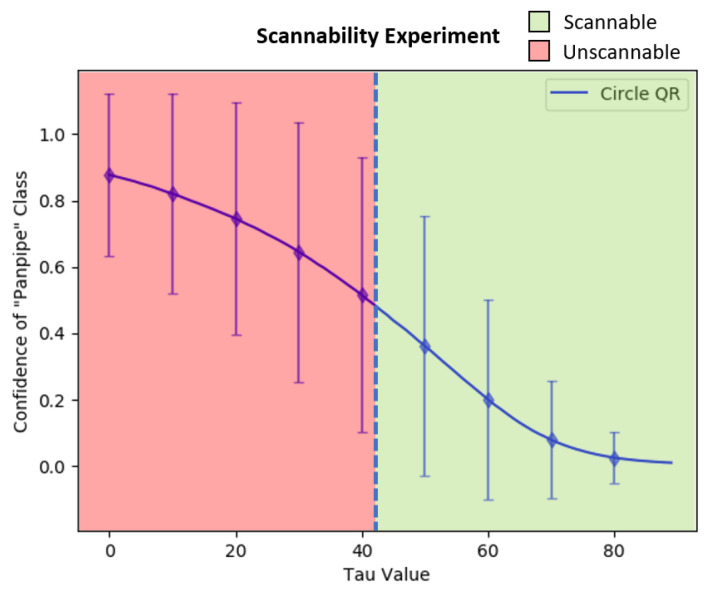
The scannability threshold experiments over the varying τ from 0 to 89 on the circular-shaped QR patch.

**Figure 18:**
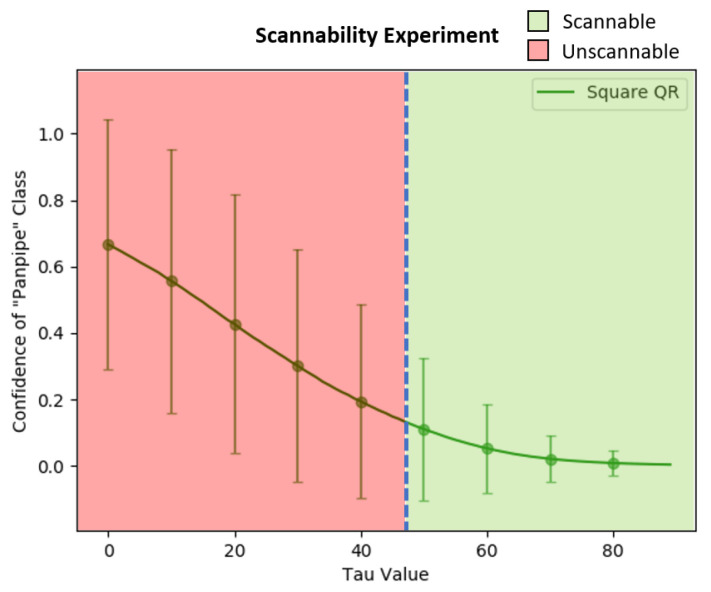
The scannability threshold experiments over the varying τ from 0 to 89 on the Square QR patch.

The results of the experiment can be seen in Figure 17 and Figure 18, in which the vertical blue dash line represents the minimal τ for the mobile phone’s QR scanner to be able to scan the QR symbol for both patch types, which were approximately 42 for the circular-shaped patch and 48 for the square patch. However, many factors can affect the patch’s scannability to a scanner, including the application used to scan, the reflection from the scanned surface, the distance between the patch and the scanning device, and the brightness of the scanning environment.

### 3.5. Class-Specific Adversarial Performance

Since the principle of the adversarial patch is to introduce a visual pattern that perturbs the saliency of an existing visual feature in an image indicating the primary category of object, the experiment in this section was performed to observe the adversarial efficacy over images of different categories and find the image patterns that were most or least influenced by the adversarial patch. In the experiments, we have applied five adversarial QR patches (Non tau-adjusted): *k*∈ {*Orangutan (#365)*, *Airliner (#404)*, *Airship (#405)*, *High bar (#602)*, *iPod (#605)*}, as shown in Table 1. The adversarial QR patches were tested over 70,000 samples that were randomly selected over 1000 classes in the ImageNet dataset.

The evaluation metric can be expressed using the following equations:

(6) $$C_{t,i,k}=f\left(x_{adv,k}^{\left(i\right)}\right)$$

where $C_{t,i,k}$ is the predicted class label using the image classifier f(·), *i* refers to an index of *N* tested images in the class $C_{t,i,k}$, and $x_{adv}$ is the randomized sample with the kth patch applied through the patch operator A(p,q,x,r,l) in Equation (3).

To evaluate the success rate of the adversarial QR patch, we can calculate:

(7) $$w_{i,k}=\left\{\begin{array}{cc} 1, & C_{s,i,k}\neq C_{t,i,k}\\ 0, & \mathrm{otherwise} \end{array}\right.$$

where $w_{i,k}$ is a binary value indicating if the attack of the *k*th adversarial QR patch is a success or failure. $C_{s,i,k}$ is the ground-truth class of the source image before adding the adversarial QR patch.

(8) $$W_{k}=\frac{1}{N}\sum_{i=0}^{N-1}w_{i,k}$$

where $W_{k}$ is the overall successful attack rate (0 to 1) given the *k*th adversarial QR patch.

Finally, the average successful attack rate of *K* types of adversarial QR patches (%) can be written by:

(9) $$Winrate=\frac{100}{K}\sum_{k=0}^{K-1}W_{k}$$

**Figure 19:**
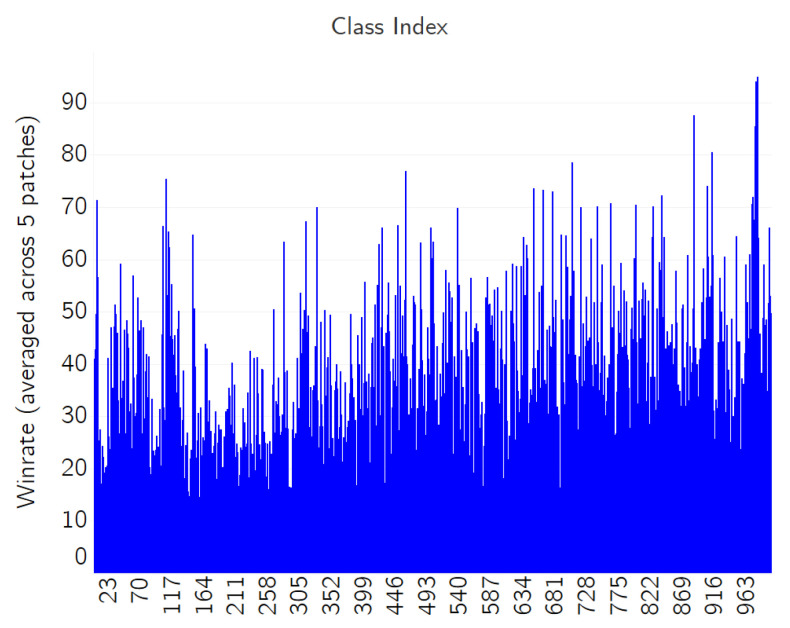
The win rate averaged across five patch attacks.

The results of the experiment are shown in Figure 19, where the vertical axis represents the average win rate over a particular class index, which is represented by the horizontal axis. Table 2 illustrates the average win rate across attacks of five different adversarial QR patches: the top five classes that are most resistant, as well as the most vulnerable, to the adversarial patch attack. From the results, it can be implied that larger, repeating patterns such as landscapes are easier to fool than the classes with well-defined, non-repeating features such as numbers on the odometer and the wing patterns of a monarch butterfly. However, the object size does not seem to have much effect on this aspect, since the images from both the Valley and the Monarch classes have the subject covering almost the whole frame.

### 3.6. Adversarial Performance Comparison against Other Learning Models

**Figure 20:**
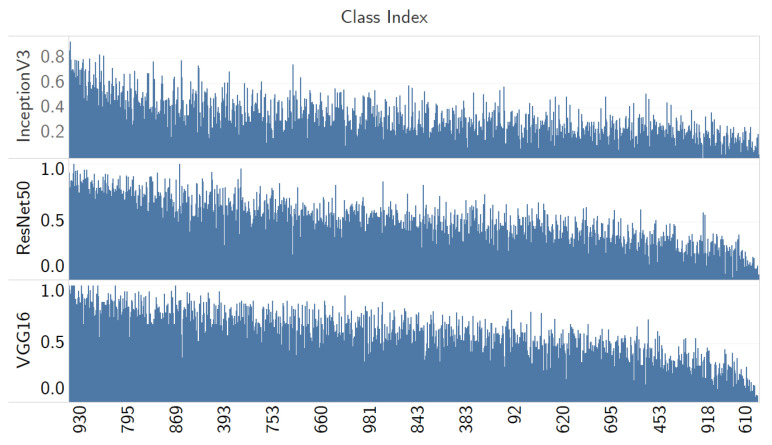
The win rate for adversarial QR patch attack (trained by InceptionV3) against three classifiers: ResNet50, VGG16 and InceptionV3; the data are sorted in a descending manner from averaging the win rate across all the three models.

Using the evaluation metrics from Section 3.5, we applied the Adversarial QR Patch of the class *Airship (#405)* trained by the InceptionV3 model on the test images. Then, we classified the test images with the pre-trained model of Resnet50 [28] and VGG16 [13] to observe the generalization of the adversarial QR patch trained by InceptionV3. To understand whether the characteristics in adversarial performance observed in the previous section also translate to other CNN models, the results are shown in Figure 20, and the data are sorted in a descending manner averaging the win rate across all the three models. The top five classes in Table 3, which are (*Valley (#979)*, *Foreland (#976)*, *Seashore (#978)*, *Velvet (#885)*, and *Lakeside (#975)* have an average win rate of (0.874, 0.873, 0.724, 0.827, and 0.776). The patch was only trained on the InceptionV3 model, yet it has worked in a similar trend when used to attack against multiple models. This exemplifies the generality of the adversarial patch as well as transferability across other different deep learning models.

## 4. Conclusions

To conclude, QR codes can be practically used as an adversarial patch against traditional image classification models while retaining their scannability, making them viable for real-world application. Despite the trend where most QR patches are created in square shapes, the experiment results revealed that the circular-shaped QR patch displayed a stronger adversarial efficacy and scannability. The most acceptable τ value used to adjust the patch’s dark parts, which maximizes the patch’s adversarial efficacy and scannability, was found to be 48 for square-shaped patches and 42 for circular-shaped QR patches. We have found that the patch trained against a particular classifier network could also be used on other models. Furthermore, we have found that image classes composed of repeatable patterns are much easier to fool than those that were not. Finally, further expansions can be made to this research in the future, including studying methods to improve the QR code’s scanning distance and angle, and time and resource optimization for the adversarial patch training process. The length of the QR code string can also be studied further to see if it has any impact on the adversarial patch’s efficacy and QR scannability.

## Figures and Tables

**Table 1:** Five QR patches generated to test per-class adversarial performance.

Patch	Class Index: Label	Sample Images
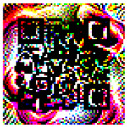	365: Orangutan	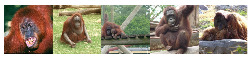
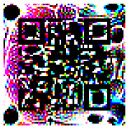	404: Airliner	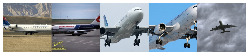
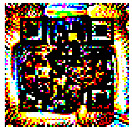	405: Airship	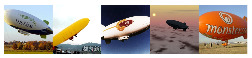
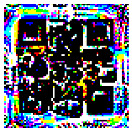	602: High bar	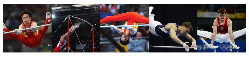
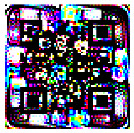	605: iPod	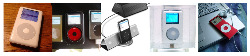

**Table 2:** Top five classes that are resistant and vulnerable to adversarial patch attacks.

Index	Class	*Winrate* (%)	Sample Images
979	Valley	95	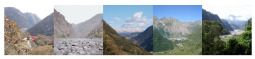
976	Foreland	94	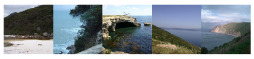
978	Seashore	89.30	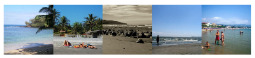
885	Velvet	87.65	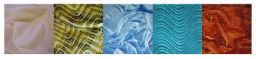
975	Lakeside	85.45	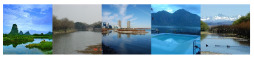
..	..	..	..
..	..	..	..
..	..	..	..
181	Bedlington terrier	7.06	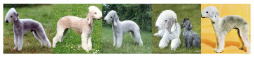
779	School bus	6.55	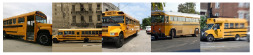
251	Dalmatian	5.97	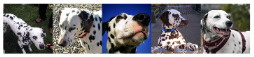
323	Monarch	5.25	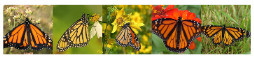
685	Odometer	4.86	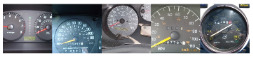

**Table 3:** Top five classes and bottom five classes that are resistant to adversarial patch attacks when averaged across three models, with previous entries from Table 2.

Rank	Index	Name	ResNet50	VGG16	InceptionV3	Average	Sample Images
1	996	Maitake	92.59	100.00	71.43	88.01	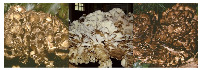
2	979	Valley	80.00	96.30	85.94	87.41	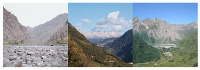
3	976	Foreland	78.05	90.63	93.10	87.26	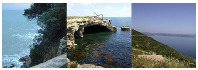
4	147	Grey Whale	85.19	93.10	78.72	85.67	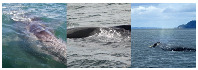
5	536	Docking	85.71	100.00	70.21	85.31	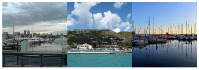
..	..	..	..	..	..	..	..
9	885	Velvet	80.00	100.00	68.18	82.73	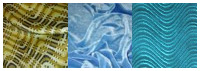
..	..	..	..	..	..	..	..
20	975	Lakeside	83.33	77.42	72.00	77.58	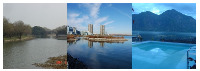
..	..	..	..	..	..	..	..
45	978	Seashore	79.17	55.56	82.69	72.47	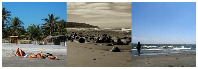
..	..	..	..	..	..	..	..
665	251	Dalmatian	65.31	44.44	12.31	40.69	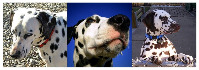
..	..	..	..	..	..	..	..
879	323	Monarch	46.67	30.16	10.00	28.94	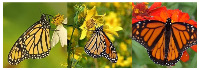
..	..	..	..	..	..	..	..
946	685	Odometer	38.10	29.17	2.74	23.33	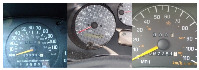
..	..	..	..	..	..	..	..
957	779	School Bus	28.30	30.77	6.90	21.99	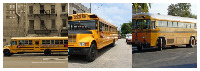
..	..	..	..	..	..	..	..
974	181	Bedlington Terrier	24.19	28.21	4.48	18.96	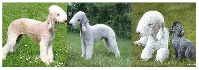
..	..	..	..	..	..	..	..
996	411	Apron	9.62	6.25	8.20	8.02	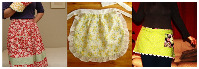
997	549	Envelope	3.28	3.45	16.39	7.71	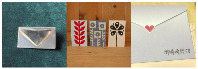
998	610	T-Shirt	12.07	5.56	2.08	6.57	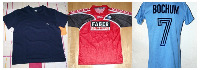
999	741	Prayer Mat	0.00	0.00	18.97	6.32	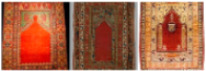
1000	721	Pillow	4.65	0.00	3.03	2.56	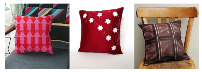

## Data Availability

Data Sharing not applicable.
